# Mechanical Properties of Composite Silty Soil Modified with Cement and Zirconia-Based Nanopowder

**DOI:** 10.3390/ma16155281

**Published:** 2023-07-27

**Authors:** Jun Hu, Chenming Xu, Junhao Ren, Hui Xiong, Zhixin Wang, Yongchang Yang

**Affiliations:** 1 College of Civil Engineering and Construction, Hainan University, Haikou 570228, China; hj7140477@hainanu.edu.cn (J.H.); xuchenming@hainanu.edu.cn (C.X.); xionghuisa@163.com (H.X.); 2 Collaborative Innovation Center of Marine Science and Technology, Hainan University, Haikou 570228, China; 3 Hainan Hydrogeological and Engineering Geological Survey Institute, Haikou 570206, China; wzx6868897wzx@163.com (Z.W.); 18807059228@163.com (Y.Y.)

**Keywords:** silty soil, cement, zirconia-based nanopowder, orthogonal test, discrete element simulation, compression properties, optimal ratios, curing age

## Abstract

This study assessed the modification effects of zirconia-based nanopowder and cement contents and curing age on the mechanical properties of silty soil. The orthogonal test design was applied to derive the best combination of each influencing factor using the lateral unconfined compressive test. Two-dimensional particle flow code (PFC^2D^) distinct-element modeling software was also used to fit and analyze the test curves, as well as simulate the triaxial test with the derived parameters. The test results reveal the optimal combination of 20% cement, 2% zirconia-based nanopowder, and 28 d curing age. The extreme difference table was used to plot the orthogonal trend diagram, and cement content was found to be the most significant factor controlling the silty soil strength. The maximum peak stress was 2196.33 kPa under the optimum combination of factors, which could be obtained through the index estimation, and these results were experimentally verified. According to the predicted strength envelope, the cohesive force of nanopowder-cement-modified silty soil in the optimal proportion was 717.11 kPa, and the internal friction angle was 21.05°. Nano zirconium dioxide will accelerate the hydration reaction of cement, the flocculent structure produced by the hydration of cement and soil particles connected to each other, play the role of filling and anchoring, and thus increase the strength of the nano-zirconium dioxide, and the optimal dosage of nano-zirconium dioxide is 2%.

## 1. Introduction

Several construction projects, including the construction of the Hainan Free Trade Port, are currently underway in the Haikou Bay located in the northern part of Hainan Island, China, which is often referred to as “China’s Hawaii” for its picturesque tropical beaches. However, the silty soil in the bay area makes such construction rather problematic, causing difficult pile formation at construction sites, insufficient strength of concrete poured on-site, and failure of the composite foundations. This particular kind of Haikou Bay silty soil is gray-black, plastic, with lustrous cut, high toughness, and slightly irritating odor. It differs from soft soil by different particle gradation and from shell sand by the different forms of organic matter present, having specific engineering properties [1,2,3].

The theoretical research on silt soil should include the characteristics and causes of silt soil, intrinsic models of silt soil, reinforcement theory of silt soil foundations, etc. The relevant results are rather limited. Since silty soil’s characteristics vary in different places, theoretical and practical research should be comprehensively combined.

Zhang et al. [4] conducted experiments on silty foam concrete and investigated the variation in the physico-mechanical indices of clayey soils. Jiang et al. [5] conducted a series of consolidation-drainage triaxial experiments on coral silt and the results showed that, for clean coral silt, the peak drained and critical state shear strengths were significantly correlated with the dry density. Wang et al. [6] investigated by triaxial tests the Mississippi River Valley chalk sand’s post-cyclic undrained monotonic shear behavior under different initial consolidation conditions. The results showed that the cyclic shear strength of the chalk varied with the initial overconsolidation ratio and the average effective consolidation pressure in agreement with previous studies, i.e., it increased with increasing OCR and decreasing σ’ c. The results showed that the cyclic shear strength of the chalk was significantly correlated with the initial overconsolidation ratio and the average effective consolidation pressure.

Since it is difficult to achieve the expected results by simply reinforcing silty soils with cement, especially Haikou Bay silty soils, perhaps due to the rich organic matter and other reasons, it is important to find a more economical and reasonable soil reinforcement method.

Pu et al. [7] investigated the use of consolidants such as lime and lime–cement mixtures for silt consolidation studies. Cui et al. [8] determined the shear strength and compressive deformation characteristics of sodium silicate-modified silt samples by direct shear and unidirectional compression tests. Sai et al. [9] prepared a new type of silt composite flexible curing agent with sintered red mud and matrix asphalt as the main materials to improve the silty soil’s mechanical properties. Zhang et al. [10] determined the optimum composition ratio of lignin–lime composite additives through a series of cyclic triaxial tests, and verified the recommended ratios of the unconfined compressive strength test to obtain the optimum additive ratios. Wang et al. [11] used microbial-induced calcium carbonate pre-precipitation technology to solidify the dredged river silt into a roadbed material and obtained the optimum additive ratio through the unconfined compressive strength test. The mechanical properties of the cured dredged river silt were analyzed by the unconfined compressive strength test, calcium carbonate content, test and microstructure test.

Particle flow code (PFC) is a numerical simulation technique of soil mechanics based on the discrete element method, which discretizes the soil into many particles and simulates the mechanical behavior of the soil by simulating the interaction between the particles. Williams et al. [12] performed compression tests and simulated indoor experiments using particle flow code. Utili et al. [13] performed uniaxial numerical simulations using two-dimensional (PFC^2D^, version 5.0) software to study and analyze clay-like materials’ fine-scale and macroscale parameters and their relationships. Belheine et al. [14] performed numerical simulation of triaxial tests, which implied that, in unconsolidated undrained shear tests, consolidated undrained drainage, or consolidation-drainage tests, strength was strongly related to the particles’ deformation and friction. The above brief survey outlined the limitations of the existing approaches and options for their possible mitigation.

Firstly, there are few studies on nanomaterials’ effect on silty soils’ curing. While the research on silty soil and consolidation agents is relatively mature, silty soil’s engineering geological spatial distribution varies with cities and regions, so it is significant to investigate soft soil’s physical index in particular localities for engineering construction.

Secondly, the nature of modified materials varies greatly, and the improvement and reinforcement methods differ among different materials. There are many research results related to considering the water content of silty soils. Still, there are relatively few studies on the effect of nanomaterials on the strength of cured soils. 

Finally, the experimental studies of silt map improvement rarely used numerical simulation to analyze the specimen’s damage pattern and crack development under pressure.

To solve the above problems, this paper carries out further research on the silty soil of the Haikou Bay of Hainan Island modified with nanopowder and cement at various ratios. To ensure that the modified silty soil meets the strength requirements of engineering practice, it uses the orthogonal test design to identify the optimal ratios of cement and nanopowder, as well as the optimal curing age. Eventually, the stress–strain curve under the optimal ratio is simulated by numerical simulation, and the crack development law under the ratio is verified.

## 2. Test Materials and Program

### 2.1. Experimental Raw Materials

The materials used in the paper were silty soil, cement, water, and nanopowder from Haikou Bay of Hainan Island, China.

#### 2.1.1. Silty Soil

The silty soil of Haikou Bay used in this study was grayish-brown in color and fluid-plastic in shape. The basic physical indices are listed in Table 1. The silty soil of Haikou Bay used in this paper was taken from the expansion project of the No. 3 teaching building of Hainan University in Meilan District, Haikou City, China, with a burial depth of 10–15 m.

#### 2.1.2. Cement

Cement used in the test was Tianya brand PO42.5 ordinary silicate cement, which was produced by Hainan Chengmai Huasheng Tianya Cement Co., Chengmai, Hainan, China. The main components of Tianya cement are given in Table 2. 

**Figure 1:**
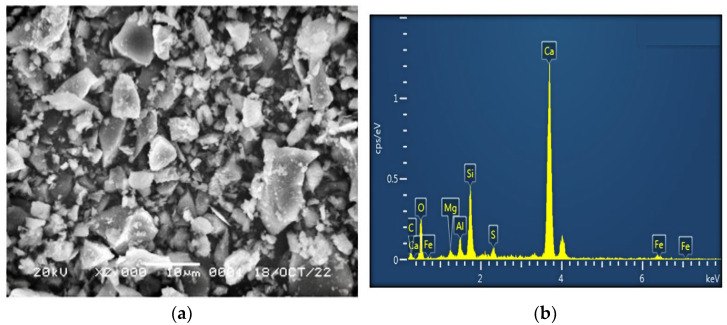
Scanning electron microscopy (**a**) and energy spectrum analysis (**b**) of cement under study.

The SEM image and EDS diagram are shown in Figure 1.

#### 2.1.3. Nanopowders

**Figure 2:**
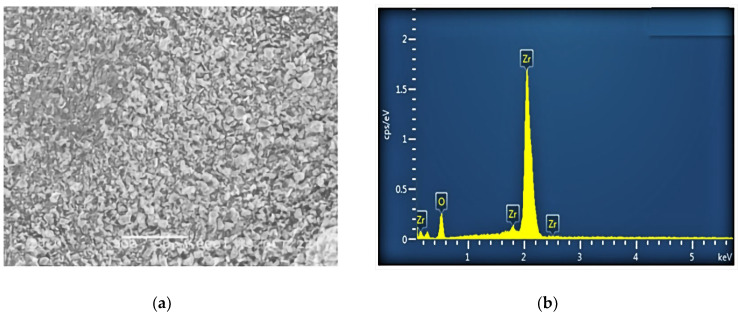
SEM image (**a**) and EDS analysis (**b**) of the nanopowder under study.

The nanopowder used in this study was produced by Zhongte Materials Co., Ltd (Anshan, Liaoning, China). Shares of its main components (zirconia, SiO_2_, Fe_2_O_3_, and TiO_2_) are summarized in Table 3. The SEM image and EDS diagram (with elemental analysis) are shown in Figure 2. Zirconia is chemically very stable and has good thermo-chemical stability and high temperature conductivity. Zirconia has high strength and toughness, and strong mechanical properties. At the same time, zirconium oxide can be used as a separate modification material, due to the small size of zirconium oxide nanopowder particles, so that its specific surface area is greatly increased, and greatly improving the modification effect.

### 2.2. Experimental Program

In the present experiment, the effects of nanopowder admixture, cement admixture, and the curing age of the silty soil on its strength were considered. The water content was controlled in the experiment, and the effects of different admixtures of cement (10%, 15%, and 20%) and nanopowder (0%, 1%, 1.5%, and 2%) on the compressive properties of silty soils at different ages (7 d, 14 d, and 28 d) were investigated. Considering a large number of influencing factors in the silt soil modification experiments, the orthogonal analysis [15,16,17,18] was applied, as shown in Table 4.

### 2.3. Preparation and Curing of Specimens

**Figure 3:**
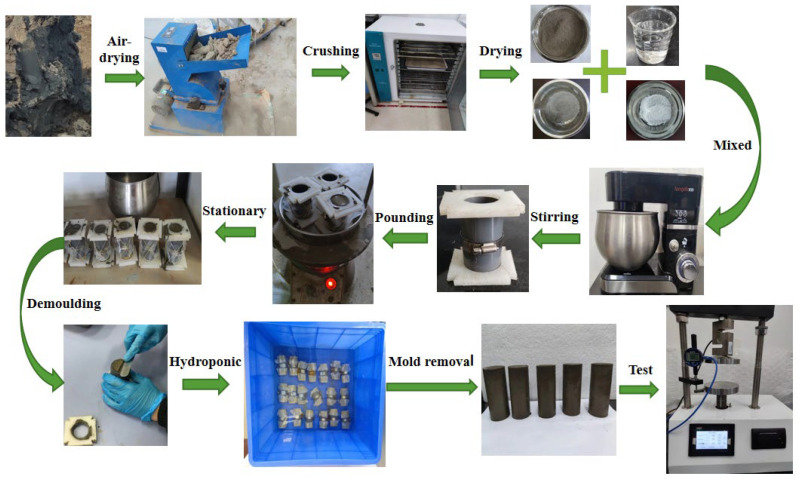
Preparation process of modified soil specimens.

The specimens for unconfined lateral compressive tests were cylinders with diameter *D* = 39.1 mm and height *H* = 80 mm. The specific operation steps for making silty soil specimens are shown in Figure 3:

(1) Pre-preparation: Before sample preparation, soil was air-dried and dried to ensure a zero-moisture content. Then, the samples were passed through a 2 mm sieve to remove impurities.
(2) Mold preparation: Molds were cleaned and dried. Then, petroleum jelly was applied evenly on the inside of the molds, which were assembled by tying them with leather straps.
(3) Preparation of soil samples: Silty soil, cement, water, and nanopowder were weighed separately. The cement and nanopowder were first put into the mixing pot of silt soil and stirred evenly by hand. Then, the mixing pot was placed on the mixing machine for stirring, and the weighed water was slowly poured into the mixing pot during the stirring process. Low- and high-speed stirring modes were used during mixing, and the mechanical stirring time exceeded 10 min.
(4) Specimen forming: The mold was filed with well-mixed modified soil in three portions, each time with sufficient shaking to exclude air.
(5) Resting and demolding: The soil sample was left in the mold for 2 h and the lid was removed. A spatula was used to scrape off the outside of the mold, and then the ends of the sample box were wrapped with filter paper and secured with a leather strap.
(6) Conservation: The specimens were placed in water to stand. The water surface should be higher than 2 cm above the specimen, and there should be appropriate intervals between specimens.
(7) Demolding: The specimens of the required curing age were removed from the mold using a counterforce frame to make a specimen 39.1 mm in diameter and 80 mm in height.
(8) Inspection: The weight of the specimen was measured using a balance, and the height of the specimen was measured using a Vernier caliper. After the test, the mass of the modified soil specimen was approximately 165 g. The specimens exceeding the mass by ±5 g and the height by ±1 mm should be regarded as invalid specimens and re-prepared, cured, and verified again.

## 3. Conclusions and Analysis

### 3.1. Mechanical Properties of Cement-Modified Silty Soil

To investigate the strength of silty soil in Haikou Bay under varying cement content and curing age, the water content was kept constant at 50%, three cement contents were used, and the curing ages were 7 d, 14 d, and 28 d. Five specimens were made for each test (further indicated as specimens 1 to 5), respectively. Their unconfined compressive test results are listed in Table 5.

**Figure 4:**
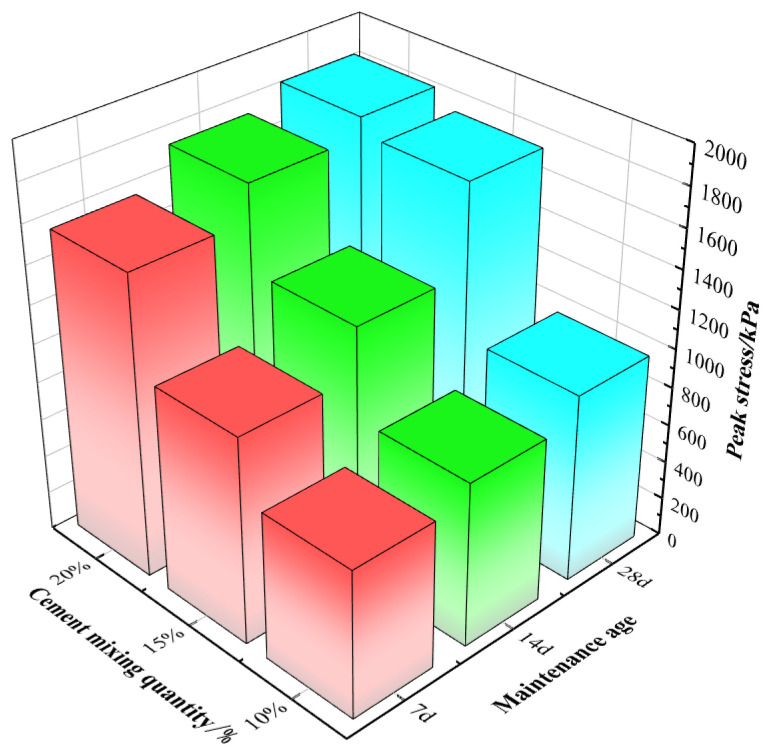
Effect of cement dosage and age of curing on peak stress of cement-modified silt.

The average values of their peak stresses are presented in Figure 4. The strength of the cement-modified silty soil slowly increased with the curing age while keeping the cement mixture constant at 10%, and increased by 12% and 10%, respectively; the strength of the cement-modified silty soil sharply increased with the curing age while keeping the cement mixture constant at 15%, and increased by 12% and 13%, respectively; the strength of the cement-modified silty soil continued to increase slowly with the curing age while keeping the cement mixture constant at 20%, and the strength of the cement-modified silty soil continued to increase with the curing age, and increased by 11 and 10%, respectively. The strength of cement-modified silty soil continued to grow gradually with curing age and increased by 11% and 10%, respectively. This was because a 10% cement content was insufficient to react fully, and that of 20% was excessive for rapid reaction.

The effects of cement dosing on cement-modified silty soil were investigated at the same curing age. The strength of the cement-modified silty soil increased rapidly with the cement content while keeping the curing age constant at 7 d. The cement-modified silt soil strength increased sharply with curing age growth from 7 d to 14 d and 28 d by 53% and 33%, respectively. At a cement content of 20%, its strength increased slowly with the curing age increased to 14 d and 28 d by 79% and 5%, respectively. The strength of cement-modified silty soil continued to increase slowly with increasing curing age and increased by 79% and 5%, respectively. The reason was that curing from 7 d to 14 d was insufficient for a complete reaction, and that from 14 d to 28 d it was twice longer, so the growth rate was lower.

### 3.2. Nanopowder-Cement-Modified Silty Soil Orthogonal Test

An orthogonal experiment is a method of designing experiments, the basic principle of which is to decompose complex experimental factors into several basic factors, and each factor is considered separately to derive experimental results. The characteristic of orthogonal experiments is that they can make full use of experimental data, reduce the number of experiments as much as possible, and eliminate the interference between factors to ensure the accuracy of the experimental results. Taking the effect of age, cement admixture, and nanopowder admixture on the strength of silty soil as an example, in the single-factor analysis, only the local level of the factors to be studied is considered, which cannot fully reflect the influence of the factors, and too many test groups are needed. In contrast, the orthogonal analysis has a balanced distribution of test points. It is evenly matched between factors, eliminating the possible errors caused by non-equilibrium dispersion and facilitating the comparison of horizontal effects.

In this paper, the data were analyzed using the visual analysis method, i.e., the polar difference method. The “extreme difference” is a way to visualize the influence of the factors in the test, expressed numerically; the greater the value of the extreme difference, the greater the influence of the factor and vice versa [19,20,21].

In this experiment, an L9 (33) orthogonal table, i.e., a three-factor, three-level orthogonal table (as in Table 6), was used to perform five replicate observation tests of its general model and its test results (*y_ij_*). In this analysis, factor A is the cement content, factor B is the nanopowder content, and factor C is the curing age.

### 3.3. Orthogonal Results and Analysis

**Figure 5:**
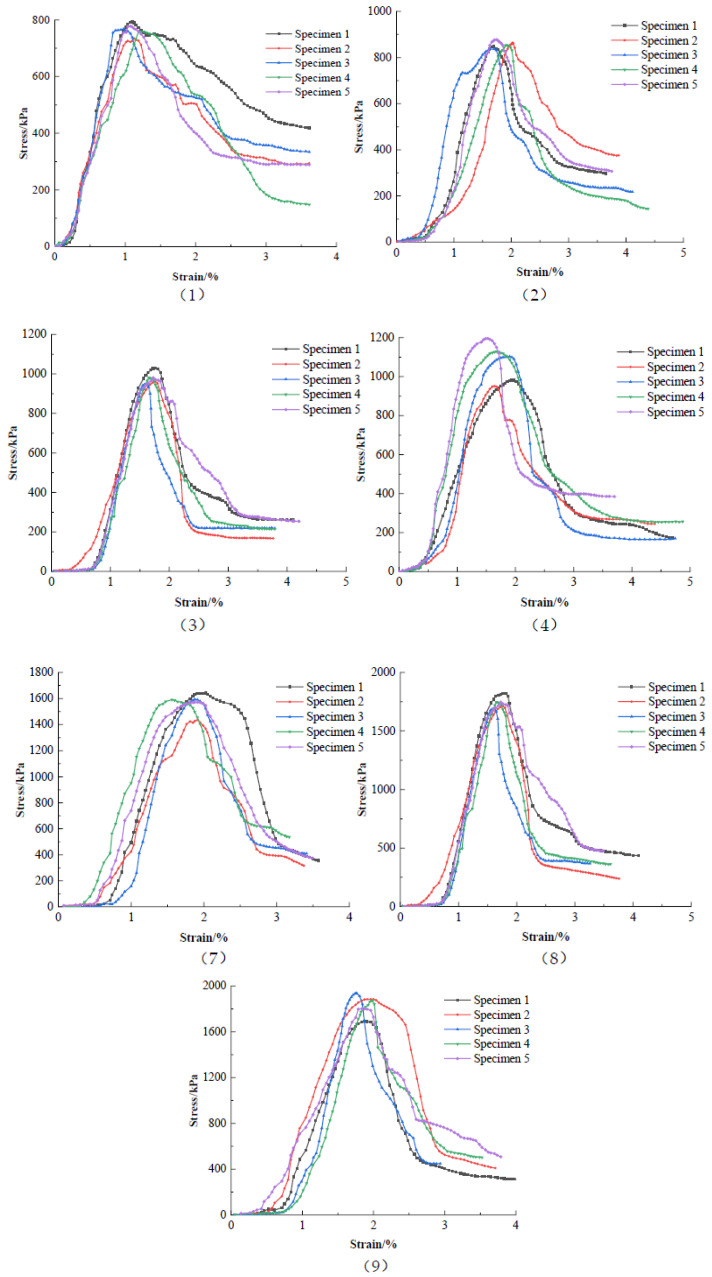
Stress–strain curves of nanopowder-cement-modified silty soil for each group of orthogonal tests.

To investigate the strength of the silty soil in Haikou Bay with different cement and nanopowder contents and curing ages, the water content was kept constant at 50%, and the cement admixture was 10%, 15%, and 20%; the curing ages were 7 d, 14 d, and 28 d. Five specimens were made for each test and referred to as specimens 1 to 5, respectively. The orthogonal tests were performed according to the orthogonal design shown in Table 6, and the stress–strain curves of the unconfined compressive tests are shown in Figure 5.

The stress–strain curves of nanopowder-cement-modified silty soil were softening-type curves, and their strains at fracture were about 2%. According to the results on cement-modified silty soil in Section 3.1., nanopowder improved its strength by approximately 10%. Its basic properties improved with increasing cement content and curing age.

### 3.4. Orthogonal Test Extreme Difference Analysis and Optimal Ratio

According to the test results, the cement content, nanopowder content, and curing age were taken as influencing factors, and the peak stress of the test data was taken as the observed value. The orthogonal test extreme difference analysis table is presented in Table 7.

From Table 7, it can be seen that *R*_1_ > *R*_3_ > *R*_2_ and the primary order of the factors was determined as A, C, and B. The optimal level was selected as level 3 (20% cement) for the largest *k*_3_ in factor A; the optimal level was selected as level 2 (2% nanopowder) for the largest *k*_2_ in factor B; and the optimal level was selected as level 3 (28 d) for the largest *k*_3_ in factor C. The optimal level combination was A_3_B_2_C_3_, i.e., a 20% cement content and a 2% nanopowder content at a 28 d curing age, which produced the best strengthening effect [22,23,24,25].

**Figure 6:**
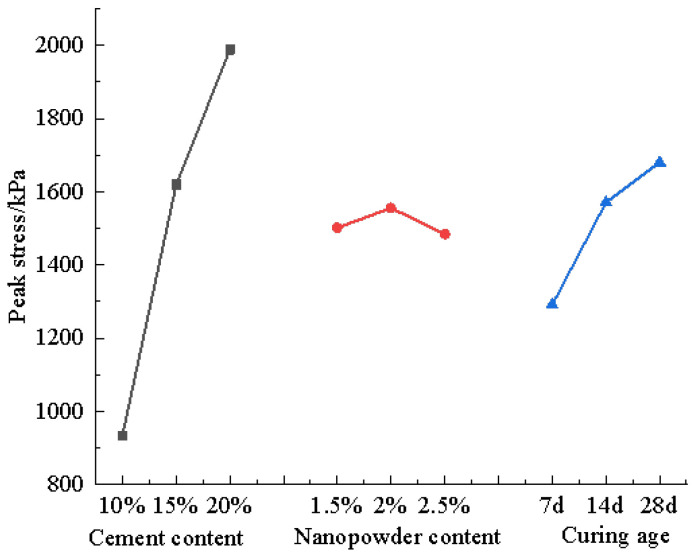
Trend graph of the orthogonal test.

Using the orthogonal test polar analysis in Table 7, the orthogonal test trend graph shown in Figure 6, the trend graph of the orthogonal test was made. It can be seen from the graph that (1) the cement content had the strongest effect on the strength of the silty soil, while the nanopowder content and curing age effects were not equal to each other. (2) With the increased cement content, the strength of the modified soil increased linearly; with the increased nanopowder content, the strength of the modified silty soil first gradually increased and then sharply dropped. With the increased curing age, the strength of the modified soil also grew. However, with increased curing age and the slow hydration reaction of cement, the strength growth gradually became slower.

*K* denotes the sum of all test values, *k* is the corresponding mean, and *R* is the extreme deviation.

$$K=\overset{9}{\underset{I=1}{\sum }}Y_{i}$$

$$k_{i}x=\frac{1}{3}K_{i}x\;\;\;\left(i=1,\;2,\;3,\;4,\;5,\;X=A,\;B,\;C\right)$$

$$R=MAX\left(K_{i}x\right)-MIN\left(k_{i}x\right),i=\left(1,2,3,4,5\right),X=\left(A,\;B,\;C\right)$$

## 4. Discrete Element Numerical Simulation Analysis

Particle Flow Code (PFC) is a general purpose, distinct-element modeling (DEM) framework that is available as two- and three-dimensional programs (PFC^2D^ and PFC^3D^, respectively). It models synthetic materials composed of an assembly of variably sized rigid particles that interact at contacts to represent both granular and solid materials. Due to the fact that there is contact/bond between the particles, a material with unique properties is simulated. Once this bond is broken, particles will separate. The particles can transmit forces (bending moments) and independently generate their displacements. Therefore, PFC can help to study the mechanical behavior of materials, including elastic deformation, plastic deformation, fracture, and damage. Generally, the particle flow method applies to the study of bulk materials. PFC has the following advantages: (1) it can simulate the microstructure inside the soil and the interaction between particles, which can reflect the mechanical behavior of the soil more realistically; (2) it can simulate the damage process of the soil, including fracture formation and expansion, which is important for the design and evaluation of geotechnical structures; and (3) it can simulate the particle fragmentation problem [26,27].

### 4.1. Basic Assumptions of PFC^2D^

This study used the two-dimensional PFC^2D^ software, with the following basic assumptions [28,29]:

(1) The particles are rigid bodies;
(2) The mutual contact between the particles is simplified by a point contact;
(3) There is an overlap between particles, and the contact force is calculated using the force-displacement law at the point of contact;
(4) Presence of bond strength is stipulated.

### 4.2. Model Build Process

In PFC^2D^, materials are represented by mutual contact in the model. Contact refers to the interaction forces that occur when particles interact with each other. However, the contact between each particle is difficult to represent, and various contact models are used to simplify this situation, namely (1) stiffness models, (2) slip models, and (3) bonding models.

**Figure 7:**
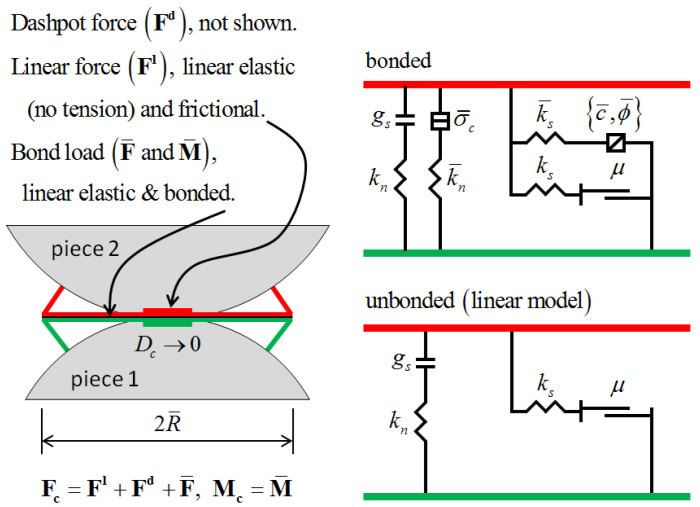
Schematic diagram of the parallel bonding model.

As shown in Figure 7, bonding models are generally used to simulate silty soils, which have mutual forces. As the name implies, there is a bonding force between the particles. There are two types of bonding models: contact bonding and parallel bonding. In the bonding model, the particles are in point contact with each other (acting on one point only), so contact bonding can only transfer forces. Parallel bonding, on the other hand, acts on a circular cross-section between two particles and is capable of transferring forces and moments. Both bonding models can be used simultaneously.

In the parallel bonding model, the contact force can be decomposed into a linear component, a bonding component, and a parallel bonding force, as follows [30]:

(1) $$\;\;\;\;\;\;\;F_{c}=F^{I}+F^{d}+\bar{F}$$

(2) $$\;M_{c}=\bar{M}$$

where **F^I^** denotes the linear force, **F^d^** is the damping force, $\bar{F}$ is the parallel bond force, and $\bar{M}$ is the parallel bond moment. The action force is decomposed into tangential and normal directions, and the moment is decomposed into bending moment and torsion (torque is 0 in PFC^2D^). The rules for calculating the action force and moment are as follows:

(3) $$F=-{\bar{F}}_{n}N_{c}+{\bar{F}}_{s}$$

(4) $$M={\bar{M}}_{t}N_{c}+{\bar{M}}_{b}\;(\mathrm{In}\;\mathrm{the}\;2D\;\mathrm{model}:\;{\bar{M}}_{t}\equiv 0)$$

The tangential forces and moments in the model appear in the cross-section where the particles are in contact with each other and are expressed in the coordinates as

(5) $${\bar{F}}_{s}={\bar{F}}_{ss}S_{c}+{\bar{F}}_{st}t_{c}\;(\mathrm{In}\;\mathrm{the}\;2D\;\mathrm{model}:\;{\bar{F}}_{ss}\equiv 0)\;$$

(6) $$\;\;\;\;\;{\bar{M}}_{b}={\bar{M}}_{bs}S_{c}+{\bar{M}}_{bt}t_{c}\;(\mathrm{In}\;\mathrm{the}\;2D\;\mathrm{model}:\;{\bar{M}}_{bt}\equiv 0)\;$$

In summary, the parallel bonded contact model was chosen in this study as the contact method for the generated particles. The parallel bonding model has the following characteristics:

(1) The parallel bonding model describes the bond strength of the material, i.e., the intermolecular forces within the material;
(2) The parallel bonding model describes the multidirectional nature of the material, i.e., the strength stiffness of the material in different directions;
(3) The parallel bonding model describes the elasticity of the material, i.e., the recovery of the original shape size of the material subjected to the load;
(4) The parallel bonding model describes the damage mode of the material, i.e., the deformation of the material when it is subjected to a load;
(5) The fine parameters should be continuously tuned to simulate the actual test results. Combined with the relevant literature, the fine parameters to be set for the parallel bonding model are listed in Table 8.

The parameters obtained from the indoor tests and the stress–strain curve criteria were used to select the set of fine parameters with the highest degree of agreement as the fine parameters for the final PFC simulation. Given a large number of fine parameters to be set for the parallel bonding model, the frequently used “trial-and-error” method was used in this paper for parameter calibration [31].

According to the dimensions of the indoor test specimens, all PO42.5-type cements were used for simulation, and the model size of PFC was also 80 mm high and 39.1 mm in diameter. The specific parameters are listed in Table 9.

### 4.3. Simulation Results and Analysis

Simulation of the test results under the optimal ratio (20% cement content, 2% nanopowder content, and 28 d curing age), the fine correlation parameters of particles were obtained and listed in Table 10.

**Figure 8:**
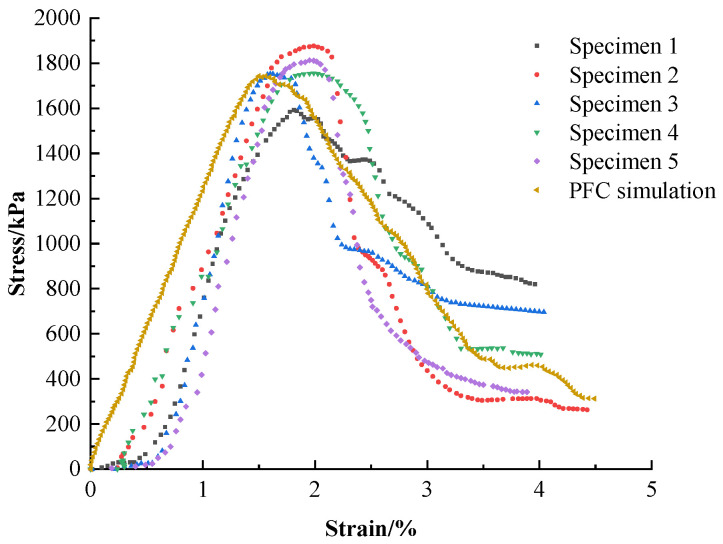
Comparison of the results of PFC simulation of the optimal proportion of nanopowder-cement-modified silty soil with experimental data.

**Figure 9:**
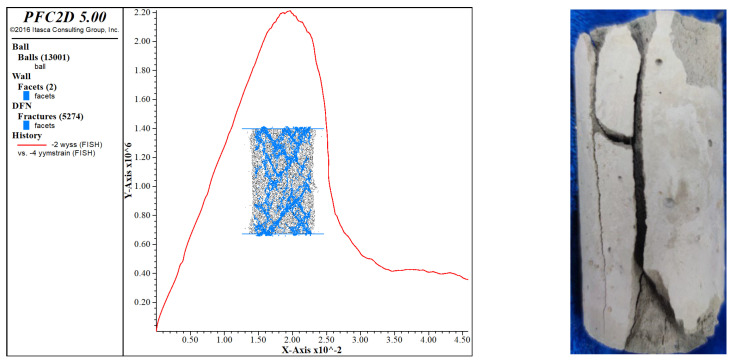
Results of PFC simulation of the optimal proportion of nanopowder-cement−modified soil.

The results simulated by PFC^2D^ are shown in Figure 8. The curves are compared in one graph by Origin drawing software, and the specific results are shown in Figure 8. The damage form of the specimen under the optimal proportion and its crack development law were also simulated and studied, and the specific results are shown in Figure 9.

The pressure phase of the PFC simulation curve grew faster than those of five specimens’ experimental curves and was roughly linear. This was due to the smaller difference between the particle set’s maximum and minimum radius values during the parameter calibration phase, resulting in less porosity between the particles. It also allowed for a complete path of force conduction between the internal particles when the specimen was compressed and with a higher stress growth rate. As the arithmetic force affected the parameter calibration, there was a reasonable error, but overall, the variation law of the actual curve was kept.

At the peak stress stage, the peak stress of the PFC simulation curve and five experimental curves coincided (2200 kPa), and the strains corresponding to the peak stress were all 1.9%. The PFC simulation and experimental data had a high agreement in terms of peak stress and peak strain, indicating that the simulation results in this stage were more satisfactory.

From the damage pattern shown in Figure 9, the test mainly showed splitting damage with bifurcated damage at the bottom, the damage simulated by PFC was mainly an internal damage pattern, and the bottom had a damage pattern similar to that of the test group.

The PFC simulated curve was generally very close to the test curve, and the parameter settings were generally correct.

### 4.4. Triaxial Test Simulation Prediction and Analysis

**Figure 10:**
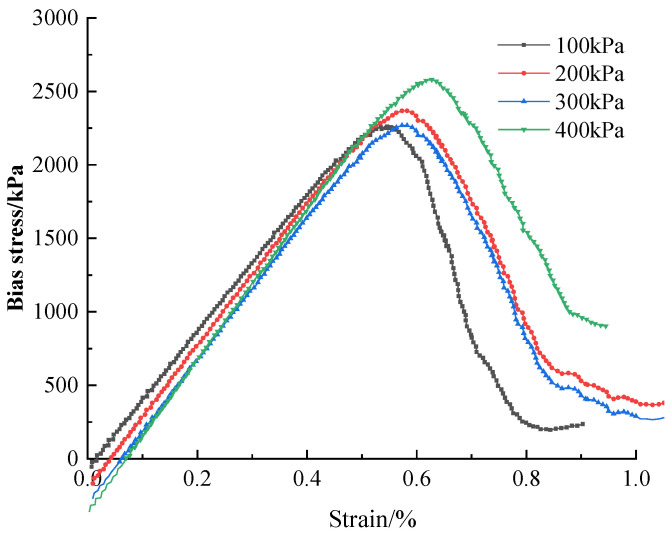
Nanopowder-cement-modified bias stress–strain curve under different surrounding pressures.

Simulation of the triaxial and unconfined compressive tests were also performed with the parameter of perimeter pressure. According to the values of the fine parameters set in the above simulation, the prediction was made by substituting unconsolidated undrained conditions into the triaxial compression model. The maximum principal stresses $\sigma_{1}$ were obtained through partial stresses $\sigma_{1}-\sigma_{3}$ for damage at different surrounding pressures (100, 200, 300, and 400 kPa). The partial stress–strain curves were plotted in Figure 10.

It should be noted that the predicted results of the triaxial test simulations are only for prediction, and the prediction tests are used to determine the accuracy of the simulations and to ensure that the experimental data are accurate, and a combination of the two is needed for the final practical application.

**Figure 11:**
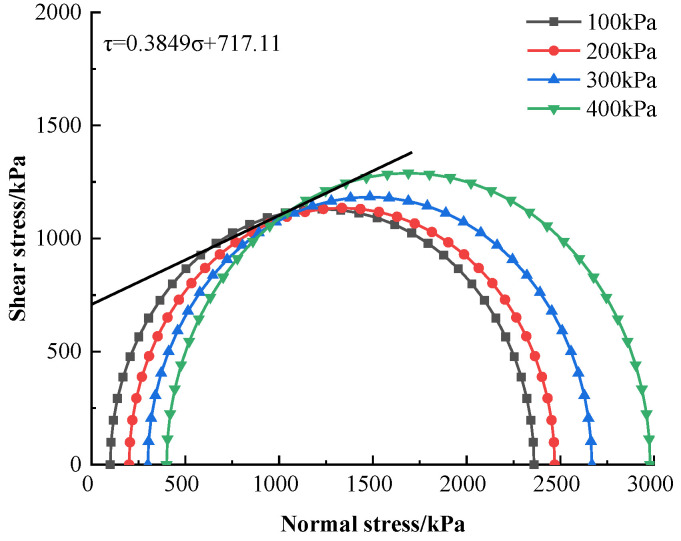
Strength envelope of optimally prepared nanopowder-cement-modified silty soil.

The shear strength index of soil is mainly measured by cohesion and the internal friction angle, both of which can be reacted by molar stress circles with semi-circle radii $\sigma_{1}+\sigma_{3}$ and $\sigma_{1}-\sigma_{3}$. The molar stress circles and strength envelopes under different enclosing pressures were plotted in Figure 11.

The cohesive force C at the optimal combination of factors was 717.11 kPa, the internal friction angle was 21.05°, and the strength curve was linearly regressed as τ = 0.3849σ + 717.11. From the obtained envelope, the variation in cohesion exceeded the variation in the internal friction angle. This change is analyzed from the principle of cement-modified soil: as the cement content increases, the amount of hydration products increases, the cementation between the particles increases, and the cohesion is improved; the hydration products fill the particle gap, and the internal friction angle increases. Therefore, cohesion and internal friction angle grow with increasing cement content, and the effect of cohesion on strength is stronger than that of internal friction angle.

## 5. Conclusions

Based on the lateral unconfined compressive tests, the strength variation in modified silty soil with different contents of cement and zirconia-based and three various curing ages was tested, and the optimal combination of factors was obtained and verified by the orthogonal test design. The damage process and crack development of the specimens were simulated by the commercial PFC^2D^ software package. The following conclusions were drawn:

(1) By orthogonal test design, the effect of zirconia-based nanopowder content (1.5, 2, and 2.5%) on cement-modified silty soil strength improvement was studied.
(2) The optimum combination of factors was A_3_B_2_C_3_, i.e., a 20% cement content, a 2% nanopowder content, and a 28 d curing age. The orthogonal trend diagram was plotted via the extreme difference analysis. It implied that cement content was the most significant factor controlling the modified silty soil strength. The maximum peak stress was 2196.33 kPa under the optimum combination of factors, which could be obtained through the index estimation, and these results were experimentally verified.
(3) According to the predicted strength envelope, the cohesive force of the optimally prepared nanopowder-cement-modified silty soil was 717.11 kPa, and the internal friction angle was 21.05°.

## Figures and Tables

**Table 1:** Physical properties of silty soil in Haikou Bay.

Specific Gravity	Water Content/%	Fluid Limit/%	Restriction on Plastic/%	Liquid Limit Index	Plastic Limit Index
2.70	49.6	44.9	24.3	1.19	21.2

**Table 2:** Main components of Tianya brand cement.

Cement Composition	CaO	SiO_2_	Al_2_O_3_	Fe_2_O_3_	FeO	SO_3_	K_2_O	Na_2_O
Content/%	65.1	20.6	4.7	3.4	2.5	2.1	0.5	0.7

**Table 3:** Nanopowder composition.

Nanopowder Composition	ZrO_2_	SiO_2_	Fe_2_O_3_	TiO_2_
**Content/%**	85	5	5	5

**Table 4:** Design of nanopowder-cement-modified Haikou Bay silty soil in lateral unconfined tests.

Specimen Number	Soil Material	Water Content/%	Cement Mixing Proportion/%	Nanopowder Doping/%	Curing Age/d
1	Silty soil	50	10	1	7
2		50	10	1.5	14
3		50	10	2	28
4		50	15	1	14
5		50	15	1.5	28
6		50	15	2	7
7		50	20	1	28
8		50	20	1.5	7
9		50	20	2	14

**Table 5:** Peak stress range of cement-modified silty soils with different cement contents and curing ages.

	10% Cement Mixing	15% Cement Mixing	20% Cement Mixing	Curing Age
**Cement-modified silty soil peak stress/kPa**	735~794	951~1196	1426~1641	7 d
	836~877	1261~1391	1688~1818	14 d
	949~1029	1602~1876	1689~1938	28 d

**Table 6:** L9 (33) orthogonal tables.

Test Number	Factor A	Factor B	Factor C	Remarks
1	A1	B1	C1	10% cement, 1% nanopowder, 7 d
2	A2	B2	C2	10% cement, 1.5% nanopowder, 14 d
3	A3	B3	C3	10% cement, 2% nanopowder, 28 d
4	A4	B4	C4	15% cement, 1% nanopowder, 7 d
5	A5	B5	C5	15% cement, 1.5% nanopowder, 14 d
6	A6	B6	C6	15% cement, 2% nanopowder, 28 d
7	A7	B7	C7	20% cement, 1% nanopowder, 7 d
8	A8	B8	C8	20% cement, 1.5% nanopowder, 14 d
9	A9	B9	C9	20% cement, 2% nanopowder, 28 d

**Table 7:** Extreme difference analysis.

Test Number	A	B	C	$y_{i1}$	$y_{i2}$	$y_{i3}$	$y_{i4}$	$y_{i5}$	$\overset{–}{y_{i}}$
1	1	1	1	791	818	809	791	852	812.2
2	1	2	2	939	964	936	860	922	924.2
3	1	3	3	1025	1088	1062	1067	1083	1065.0
4	2	1	2	1701	1636	1615	1625	1712	1657.8
5	2	2	3	2010	1904	1823	1999	1970	1941.2
6	2	3	1	1197	1278	1309	1217	1295	1259.2
7	3	1	3	1903	1956	2162	2079	2073	2034.7
8	3	2	1						1801.0
9	3	3	2						2128.8
*K*_1_	2801.4	4504.6667	3872.4						13,624.1
*K*_2_	4858.2	4666.4	4710.8						
*K*_3_	5964.46	4453	5040.867						
*k*_1_	933.8	1501.56	1290.8						1513.79
*k*_2_	1619.4	1555.47	1570.27						
*k*_3_	1988.16	1484.33	1680.29						
*R*	1054.36	71.13	389.49						

**Table 8:** Parallel bonding model’s parameters.

Particle FINE Parameters	Parallel Bonding Model Parameters
Particle porosity	Parallel contact modulus
Maximum particle radius	Parallel bonding stiffness ratio
Minimum particle radius	Normal bonding strength
Particle friction coefficient	Tangential bond strength
Linear stiffness ratio	Internal friction angle

**Table 9:** Fine-off parameters of simulated particles.

Particle Porosity	Minimum Particle Radius	Maximum Particle Radius	Friction Coefficient
0.16	0.2	0.3	0.5

Note: Particle porosity: In the granular material layer, the ratio of the void volume between particles to the volume of the entire granular material layer is called void ratio. Minimum particle radius: refers to the model set the radius of the largest particles and the minimum particle radius. Friction coefficient: The coefficient of friction is the ratio of the friction force between the surfaces of two particles and the vertical force acting on one of the surfaces.

**Table 10:** Fine parameters of simulated particles of nanopowder-cement-modified silty soil with the optimal cement and nanopowder contents and curing age.

Linear Contact Modulus/Pa	Contact Stiffness Ratio	Parallel Contact Modulus/Pa	Cohesion/Pa	Tensile Strength/Pa
10^6^	1.5	10^6^	1.23 × 10^6^	2.4 × 10^6^

Note: Linear contact modulus, parallel contact modulus: a fine-grained parameter in the linear-parallel bonded contact model. $e=\frac{\sigma }{\varepsilon }$ ε—Representative strain σ—representative stress. Contact stiffness ratio: the ratio of contact stiffness coefficient is the ratio of tangential contact stiffness coefficient and normal contact stiffness coefficient at the contact point. The cohesion, also called cohesion, is the mutual attraction between neighboring parts within the same substance, and this mutual attraction is a manifestation of the existence of molecular forces between molecules of the same substance. The cohesive force is obtained by subtracting the friction strength from the total shear strength in the case of effective stress. Tensile strength: refers to the maximum tensile stress that the model particles are subjected to at the time of breakage.

## Data Availability

Not applicable.
